# Efficacy of an Interdisciplinary Intensive Outpatient Program in Treating Combat-Related Traumatic Brain Injury and Psychological Health Conditions

**DOI:** 10.3389/fneur.2020.580182

**Published:** 2021-01-18

**Authors:** Thomas J. DeGraba, Kathy Williams, Robert Koffman, Jennifer L. Bell, Wendy Pettit, James P. Kelly, Travis A. Dittmer, George Nussbaum, Geoffrey Grammer, Joseph Bleiberg, Louis M French, Treven C. Pickett

**Affiliations:** ^1^National Intrepid Center of Excellence, Walter Reed National Military Medical Center, Bethesda, MD, United States; ^2^Department of Neurology, Uniformed Services University of the Health Sciences, Bethesda, MD, United States; ^3^Credence Management Solutions, Vienna, VA, United States; ^4^Psychological Health Center of Excellence, J9, Defense Health Agency, McClean, VA, United States; ^5^Department of Neurology, University of Colorado School of Medicine, Marcus Institute for Brain Health, Aurora, CO, United States; ^6^Booz Allen Hamilton, McClean, VA, United States

**Keywords:** traumatic brain injury, psychological health, post-traumatic stress disorder, integrative medicine, military health, intensive outpatient program, interdisciplinary care, creative arts therapies

## Abstract

**Background:** Since 2000, over 413,000 US service members (SM) experienced at least one traumatic brain injury (TBI), and 40% of those with in-theater TBIs later screened positive for comorbid psychological health (PH) conditions, including post-traumatic stress disorder (PTSD), depression, and anxiety. Many SMs with these persistent symptoms fail to achieve a recovery that results in a desirable quality of life or return to full duty. Limited information exists though to guide treatment for SMs with a history of mild TBI (mTBI) and comorbid PH conditions. This report presents the methods and outcomes of an interdisciplinary intensive outpatient program (IOP) in the treatment of SMs with combat-related mTBI and PH comorbidities. The IOP combines conventional rehabilitation therapies and integrative medicine techniques with the goal of reducing morbidity in multiple neurological and behavioral health domains and enhancing military readiness.

**Methods:** SMs (*n* = 1,456) with residual symptoms from mTBI and comorbid PH conditions were treated in a 4-week IOP at the National Intrepid Center of Excellence (NICoE) at Walter Reed National Military Medical Center (WRNMMC). The IOP uses an interdisciplinary, holistic, and patient-centric rehabilitative care model. Interdisciplinary teams provide a diagnostic workup of neurological, psychiatric, and existential injuries, and from these assessments, individualized care plans are developed. Treatment response was assessed using the Neurobehavioral Symptom Inventory (NSI), PTSD Checklist—Military Version (PCL-M), Satisfaction With Life Scale (SWLS), Patient Health Questionnaire-9 (PHQ-9), Generalized Anxiety Disorder-7 (GAD-7), Epworth Sleepiness Scale (ESS), and Headache Impact Test-6 (HIT-6) and administered at admission, discharge, and at 1, 3, and 6 months post-discharge.

**Findings:** Following treatment in the IOP, the symptomatic patients had statistically significant and clinically meaningful improvements across all outcome measures. The largest effect size was seen with GAD-7 (*r* = 0.59), followed by PHQ-8 (*r* = 0.56), NSI (*r* = 0.55), PCL-M (*r* = 0.52), ESS (*r* = 0.50), SWLS (*r* = 0.49), and HIT-6 (*r* = 0.42). In cross-sectional follow ups, the significant improvements were sustained at 1, 3, and 6 months post-discharge.

**Interpretation:** This report demonstrates that an interdisciplinary IOP achieves significant and sustainable symptom recovery in SMs with combat-related mTBI and comorbid PH conditions and supports the further study of this model of care in complex medical conditions.

## Introduction

During Operation Enduring Freedom (OEF), Operation Iraqi Freedom (OIF), and Operation New Dawn, over 2.7 million US service members (SMs) were deployed worldwide. Most of these SMs were engaged in combat operations and intensive combat training that resulted in ~17% suffering a traumatic brain injury (TBI) (1). From 2000 through the first quarter of 2019, more than 413,000 SMs worldwide were diagnosed with TBI, and of these, more than 80% were categorized as mild TBI (mTBI) (2). Although many individuals who suffer an mTBI recover uneventfully, a substantial minority report persistent post-concussive symptoms (PCS). The rates of persistent PCS are much higher among those with complicating psychological health (PH) conditions such as post-traumatic stress disorder (PTSD) and in military populations with combat-related injuries (3–5). A history of combat-related TBI represents a significant risk for developing behavioral health (BH) conditions, with more than 40% of these SMs showing evidence of PTSD, major depressive disorder, or anxiety disorders as compared to ~9% of SMs without a combat-related TBI (6, 7). Exposure to TBI and operational stressors and sustaining life-threatening polytraumatic injuries place SMs at a greater risk for long-term functional sequelae that affect return to full duty and overall quality of life (8). Conventional specialty referral-based approach in many of these chronic clinically complex active duty patients results in fragmented care and reduced efficiency in achieving the desired recovery. Strategies for a more comprehensive and holistic approach are needed to improve and return to duty and achieve favorable outcomes in multiple neurological and BH domains in military personnel with the “invisible wounds of war.”

Studies of TBI in the military have demonstrated significant and sometimes persistent symptoms even after mTBI (4, 9–12). In general, persistent PCS are thought to fall into three broad categories: somatic (including vestibular symptoms and headache), cognitive (including sleep disturbance, attention, and memory complaints), and emotional (including post-traumatic stress, emotional regulation, and depression) (13–15). Interventions designed to address just one domain have had varying levels of success (16). The prevalence of sleep disorders in patients with a history of mTBI is reported at >75% (15, 17), and post-concussive headache is reported in nearly half of service members with combat mTBI (18). These are contributing factors in persistent PCSs and can confound the recovery of other symptoms if not addressed early. In active duty military and veteran populations, a number of studies have examined the value of an integrated approach, combining efforts targeting multiple symptoms through cognitive rehabilitation, psychotherapy, and psychoeducational and integrated behavioral health integration to treat those with a history of TBI and PH issues (19–22). In the Department of Veterans Affairs (VA) care system, the Polytrauma Transitional Rehabilitation Programs (PTRP) have shown benefit in improving functional outcomes in individuals with TBI of mixed severity and comorbid PH issues. The PTRP treatment paradigm relies heavily on remediation of deficit areas and development of compensatory skills, such as memory strategy training, metacognitive strategy training for executive dysfunction, and practice of skills related to social communication (19). Other VA intensive residential programs for mTBI and PH conditions have focused heavily on treating the BH portion of the symptom profile (23), with several studies showing improvements in PCS, mood, or post-traumatic stress symptoms with such an approach. Chard et al. (20) reported on a treatment program that combined cognitive rehabilitation and cognitive processing therapy with educational groups focused on factors like nutrition, reduction of self-defeating behaviors, anger management, spirituality, and stress tolerance for those with TBI of mixed severity. While symptoms of post-traumatic stress declined in the sample overall, those with mild TBI showed less improvement than those with more severe TBI. In the same care setting, individuals with TBI and PTSD showed reductions in PCS concurrent with reduced post-traumatic stress symptoms (24). The Home Base program in Boston, Massachusetts reports a comprehensive program with tracks for those with significant post-traumatic stress or a history of TBI (21). In the PTSD track, the participants engage in daily psychotherapy groups, multiple skills groups based on dialectical behavioral therapy, to improve their interpersonal skills and emotion regulation during the 2-week program. They also attended six sessions of Resilient Warrior, a program adapted from the Relaxation Response Resiliency Program, that focuses on psychoeducation, as well as several complementary integrative health sessions, including art therapy, yoga, Tai Chi, fitness, and nutrition. The participants reported significant improvements in multiple functional domains (22).

Overall, the preliminary studies were promising and recommended large studies utilizing symptom-specific outcome measures in multiple domains for complex comorbid SM populations with longitudinal follow-up to test the durability of the treatment effects.

Endeavoring to address these gaps and enhance the care offered in the Department of Defense, the National Intrepid Center of Excellence (NICoE), the TBI Directorate at Walter Reed National Military Medical Center in Bethesda, Maryland was designed and built in 2010 as a proof-of-concept to assess the efficacy and sustainability of an interdisciplinary intensive outpatient program (IOP) in the treatment of SMs suffering with persistent symptoms from combat- and mission-related mTBI and co-morbid PH conditions. The program's aim is to place those SMs who had plateaued in their recovery and were deemed unlikely to experience additional symptom improvement on a trajectory to return to full duty. The care model is a 4-week interdisciplinary, holistic IOP that uses traditional rehabilitation, and neurological and BH treatments combined with integrative medicine interventions and skills-based training. The IOP uses a co-localized 17-discipline team to expedite diagnostic evaluation that leverages each specialty team member to build on each other's expertise to achieve common goals and to develop a collaborative care plan. The patient is at the center of the care team, enhancing patient-provider rapport, enabling a more efficient identification of goals for recovery, and providing immediate feedback of response to treatment. The rehabilitative culture of the care team emphasizes patients' learning self-efficacy and self-advocacy techniques to enhance sustainable recovery beyond program discharge.

We hypothesized that the NICoE 4-week IOP would improve symptoms in SMs with mTBI and PH conditions from pre- to post-program and sustain these improvements at 1, 3, and 6 months post-discharge. To evaluate the clinical effectiveness of the program, the primary analysis consists of seven domain-specific outcome scales at admission and discharge. Durability was assessed using the same self-report scales in cross-sectional follow-up at 1, 3, and 6 months after the SMs returned to their duty station. This study takes the next step in assessing interdisciplinary integrative medicine programs by measuring multi-domain outcomes in the real-world application of an IOP and collected longitudinal data to characterize the durability of health outcome improvements in a large patient population of active-duty SMs suffering from chronic co-morbid combat-related mTBI and PH conditions.

## Materials and Methods

### Participants

Active-duty SMs from all branches of the US armed services and National Guard were referred by their duty station primary care provider (PCP) or coordinating specialist to the NICoE at WRNMMC for enrollment in the 4-week IOP from August 2011 through February 2019. All enrolled patients had medically documented persistent or worsening mission- and combat-related mTBI and PH symptoms following unsuccessful treatment by multiple healthcare disciplines. All referred service members were at a minimum of 6 months post-injury, with an average of 5.09 years post-brain injury, prior to engaging in the program. TBI diagnosis was based on at least one qualifying event as specified by the Department of Defense (DoD) and VA guidelines. The NICoE Continuity Management Team, a team of social workers and nurse case managers, coordinated the assessment of eligibility with team physicians and compiled relevant clinical care administered to the SMs prior to their admission. The information was then reviewed by members of the interdisciplinary care team in advance of the initial intake interview with the SMs. The SMs remained on active duty during the IOP and attended the program voluntarily. They are supported by their commands who detail them to Walter Reed as their duty station. All SMs must have a referring physician from their duty station since the IOP generates a robust discharge summary and recommendations that are conveyed by the team to the referring provider during a warm handoff so as to maintain a smooth continuity of care.

TBI number and type were characterized as blast, non-blast, and mixed exposure to blast and non-blast events (**Table 2**). Data on TBI were obtained from a detailed intake questionnaire cross-referenced with the neurology specialist intake history and physical examination.

Up to six SMs were admitted to the NICoE IOP each Monday and navigated through the 4-week IOP as a therapeutic cohort. The SMs and their family members who attended, usually in the latter weeks of the program, resided in a dedicated living facility on campus designed to extend the NICoE's therapeutic milieu.

The SMs admitted to the IOP were invited to participate in a WRNMMC Institutional Review Board (IRB)-approved database protocol. Of these, 91.2% of patients consented, which allowed for the collection of all acquired clinical data relevant to TBI, psychological and medical comorbidities, and its storage in a coded database prior to, during, and following the program. Consent also allowed for follow-up contact of all participants through telephone contact and/or electronic questionnaires once they return to their duty station. Under an IRB-approved data use protocol, the study analyses were performed on de-identified data from the coded database in accordance with all federal laws, regulations, and standards of practice as well as those of the DoD and the departments of Army/Navy/Air Force.

### Procedures: Model of Care

The model of care in this interdisciplinary program is based on three foundational principles: (1) immediately provide a safe and trusting environment and address sleep disturbance and pain, (2) use a patient-centric approach to facilitate self-identification of the physical and existential injuries resulting in major post-concussive symptomatology and suffering, and (3) provide training and education to optimize long-term self-efficacy and self-advocacy.

**Figure 1:**
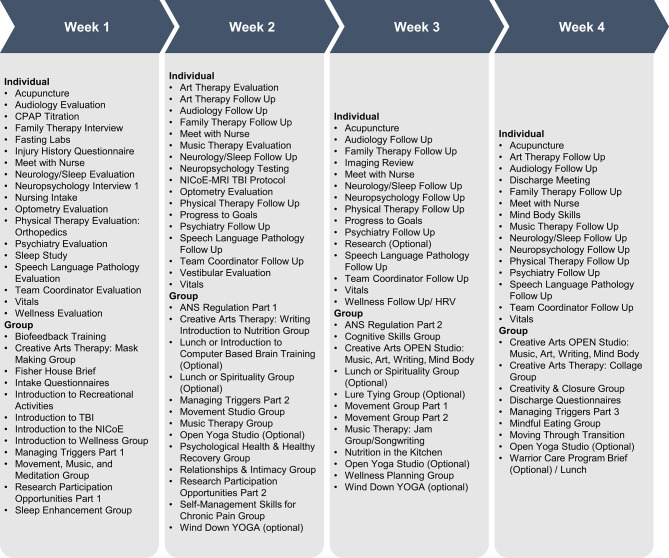
NICoE Schedule. In the NICoE patient-centric model, the clinical schedule is tailored to each service member who engages in 6–8 h of clinical care per day, totaling ~105–135 h in the 4 weeks.

Following an intensive pre-admission review of the patients' medical record, each patient engages in a group interdisciplinary intake with core team members on the first day of the program. The core team members include an internist, neurologist, psychiatrist, neuropsychologist, family therapist, and a designated nurse specialist who serves as the SM's “touchstone” throughout the program. At the center of this team is the patient, who, with their family, communicates a narrative of events that led to the physical, psycho-social, and existential injuries and shares their goals for recovery. During the first 2 weeks of the program, the patients undergo a progression of standardized evaluation of assessment tools, spanning providers from 17 disciplines who coordinate a care plan taking into consideration all relevant diagnoses. The clinical schedule is then customized to the clinical needs of each patient. A patient engages in 6–8 h per day of clinical care, totaling ~105–130 h of patient–provider contact during the program (Figure 1). The intensive time frame optimizes patient iterative feedback and allows for the rapid modification of treatment strategies by the care team. All clinical providers meet formally twice weekly in interdisciplinary team rounds to share information and updates.

Intensive evaluation and treatment of headaches and neurological, vestibular, musculoskeletal, optometric, and audiologic disturbances are initiated to address somatic complaints. Comprehensive neurocognitive assessment with cognitive rehabilitation modules, occupational therapy, and speech language pathology addresses cognitive disturbances, including the common complaints of concentration, memory, and language disturbances, and the sleep laboratory provides full sleep assessment and treatment. The program utilizes integrative medicine (IM) approaches to reach patient treatment goals of emotional regulation. The IM offerings have two main components: first, creative arts modalities including the art therapy technique of mask making, music therapy, and therapeutic writing are introduced to aid in BH assessment and treatment that assist with externalization of previously unreported existential trauma (25) and, second: mind–body techniques, including yoga, meditation, imagery, Tai Chi, nutrition, acupuncture, and animal-assisted therapy, are offered to help the patients learn self-regulation strategies to mitigate against the effects of autonomic disturbance (26). Time is available in the 3rd and 4th weeks of the program to schedule additional sessions in those IM offerings which the patients find most efficacious. An average of 30 h is spent in IM techniques during the 4-week program, including an average of 9.6 h in creative art therapy.

Up to 15 1-h patient and family educational modules are integrated into the evaluation and treatment phases of care to enhance the understanding of the disease state, improve compliance by conveying the value of treatment, and increase self-advocacy following discharge. These educational modules also include an introduction to the biological effects of TBI and operational stressors, sleep hygiene and management, nutrition, exercise triggers, and neurocognitive training (Figure 1).

At the completion of the IOP, the NICoE primary care team lead and patient engage in a “warm handoff” teleconference with the home-based PCP and nurse case manager to review a full set of findings and clinical recommendation. The PCP assumes lead on follow-up care.

### Outcomes

Symptom-specific validated outcome metrics for each key discipline were used (20). Post-traumatic symptomatology was measured through a battery of self-report assessments, including the Neurobehavioral Symptom Inventory (NSI) (27), PTSD Checklist-Military (PCL-M) (28), Satisfaction With Life Scale (SWLS) (29), Patient Health Questionnaire-9 (PHQ-9) (30), Generalized Anxiety Disorder-7 (GAD-7) (31), Epworth Sleepiness Scale (ESS) (32), and Headache Impact Test-6 (HIT-6) (33) (Table 1). The patients completed these self-report scales (SRS) at IOP admission and discharge.

**Table 1:** Assessments administered to measure the efficacy of the NICoE 4-week intensive outpatient program.

**Assessment**	**Objective/description**	**Scoring**
Neurobehavioral Symptom Inventory	Objective: to assess post-concussion symptoms Questionnaire used to measure the severity of symptoms resulting from concussions and other head injuries and often associated with post-concussion syndrome (27, 34, 35)	22 items, five-point Likert scale: from 0 (none) to 4 (very severe) Global score ranging from 0 to 88, higher score = higher severity of symptoms, symptom-based cluster scores for three- and four-factor scoring Symptomatic range: does not have a composite threshold for symptomatic classification Clinically improved: ≥5-point change
Post-traumatic Stress Disorder Checklist, Military	Objective: to assess the symptoms of post-traumatic stress disorder (PTSD) Questionnaire used to measure the 17 Diagnostic and Statistical Manual of Mental Disorders Version IV symptoms of PTSD. The PCL-M is the military version (28, 36, 37)	17 items, five-point Likert scale: from 1 (not at all) to 5 (extremely) Global score ranging from 17 to 85, higher score = higher severity of symptoms Symptomatic range: ≥35 Clinically improved: ≥10-point change
Satisfaction With Life Scale	Objective: to assess global life satisfaction Measures global judgments of one's life rather than satisfaction with specific domains (29, 38, 39)	Five items, seven-point Likert scale: from 1 (strongly disagree) to 7 (strongly agree) Global score ranging from 5 to 35, higher score = higher satisfaction Symptomatic range: ≤ 19 Clinically improved: ≥5-point change
Generalized Anxiety Disorder Scale-7	Objective: to identify probable cases of generalized anxiety disorder (GAD) and assess symptom severity in GAD Self-report questionnaire used to screen and measure the severity of generalized anxiety disorder. Patients rate the severity of seven symptoms and indicate their occurrence within the previous 2 weeks (31, 40, 41)	Seven items, four-point Likert scale: 0 (not at all), 1 (several days), 2 (more than half the days), and 3 (nearly every day) Global score ranging from 0 to 21, higher score = higher severity of anxiety symptoms Symptomatic range: ≥10 Clinically improved: ≥5-point change
Patient Health Questionnaire (PHQ)-8	Objective: for the assessment of mental disorders, functional impairment, and recent psychosocial stressors Self-report used to screen and diagnose depression, anxiety, and alcohol and eating disorders. The PHQ-8 has eight questions, whereas the PHQ-9 contains a ninth question about suicide ideation (30, 42, 43)	Eight items, three-point Likert scale Global scores ranging from 0 to 24, lower score = better QoL Symptomatic range: ≥5 Clinically improved: ≥5-point change
Epworth Sleepiness Scale	Objective: to measure a subject's usual level of daytime sleepiness or average sleep propensity Self-report questionnaire used to quantify daytime sleepiness. Patients rate their chances of dozing off or falling asleep while engaged in different activities (32, 44)	Eight items, four-point Likert scale Global score ranging from 0 to 24, higher score = higher sleepiness Symptomatic range: >10 Clinically improved: ≥2-point change
Headache Impact Test-6	Objective: to assess the impact of headaches Self-report questionnaire designed to provide a global measure of the impact adverse headaches have on normal daily life and ability to function (33, 45)	Six items, five-point Likert scale: 6 (never), 8 (rarely), 10 (sometimes), 11 (very often), and 13 (always) Global score ranging from 36 to 78, scores by items ranging from 6 to 13, higher score = greater impact on the QoL Symptomatic range: ≥50 Clinically improved: ≥8-point change

Cross-sectional assessments of long-term follow-up after SM's return to their duty station were collected through telephone interviews and/or using a custom module on the Wounded, Ill, and Injured Registry (WIIR) that provides a secure access to the electronic scale questionnaires. All patients were queried through the automated WIIR system at 1, 3, and 6 months after discharge from the IOP. All SRS data used in this analysis were collected through the WIIR system.

### Infrastructure

To effectively deliver integrative medicine treatments and assessments, a dedicated facility that employed best practices of an optimal healing environment was designed, including maximal natural light in common spaces, use of curved walls and wood tones, and creation of environments for art and music therapy, yoga, and acupuncture (26). Furthermore, the facility was designed to co-locate key disciplines to facilitate the regular discussion of patient care plans and progress through formal and informal meetings. In addition, the facility was designed with an informatics technology capability for the systematic collection of evaluation, treatment, and outcome data elements to support practice-based evidence (PBE) analysis.

### Study Design

**Figure 2:**
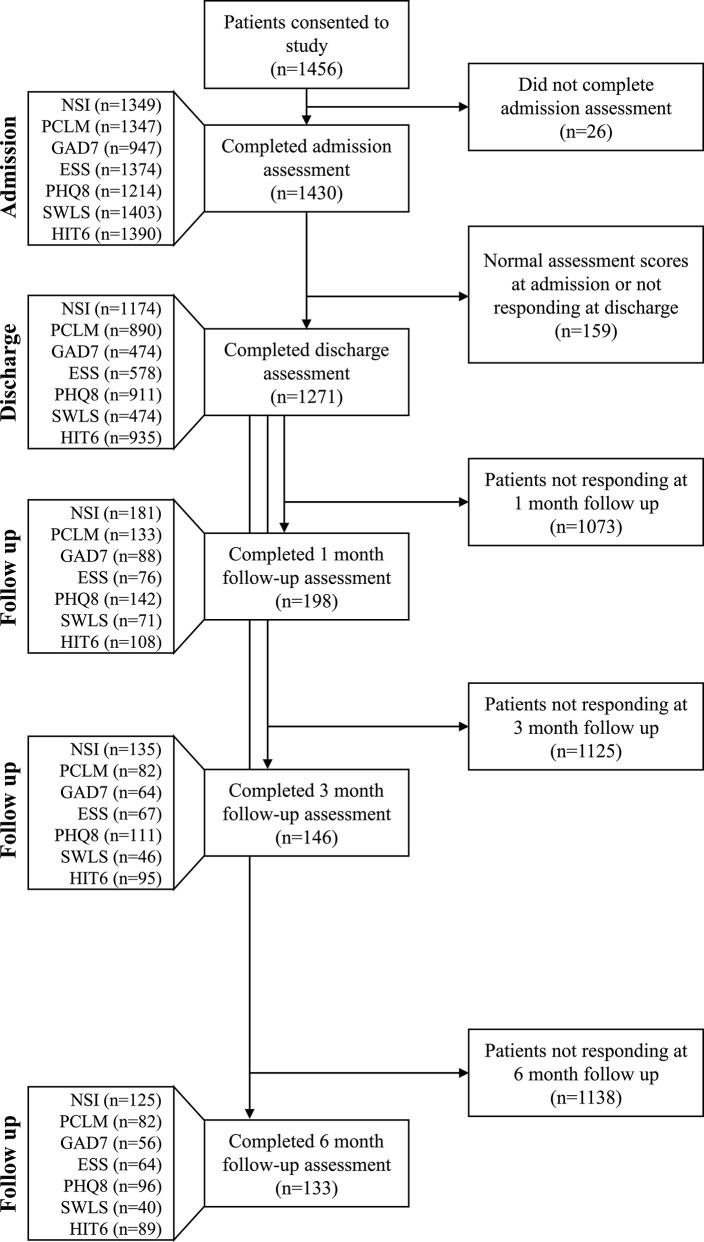
Impact of analysis criteria and response rate to follow-up questionnaires on sample size. Only patients who scored in the symptomatic range on an admission assessment and subsequently completed a discharge assessment were included in the primary analysis; PCL-M ≥35, SWLS ≤19, GAD-7 ≥10, PHQ-8 ≥5, ESS >10, and HIT-6 ≥50. The NSI does not have a composite threshold for symptomatic classification. All patient responses at any follow up time point were included in the longitudinal analysis. Sample size at each follow up time point was determined independently of the previous follow up time point (i.e., patients were included in the 3 and 6 months follow up groups regardless of whether they completed assessments for previous follow up time points).

The study followed a longitudinal design, using a pre-/post-test analysis, to assess IOP efficacy for reducing symptoms among patients with mTBI and co-morbid PH conditions. The patients with completed assessments at both admission and discharge were included in the primary analysis. For the long-term follow up, the patients were required to have completed assessments at both admission and discharge and then responded to at least one or more of the 1-, 3-, or 6-month follow-up encounters to be included in the analysis (Figure 2).

Symptom measures for all consented subjects, regardless of initial symptom severity score, were included in the analysis. Since this group would then include patients who had never had those symptoms or condition, we performed a response-to-therapy analysis for patients who met the following symptom severity thresholds—PCL-M ≥35 (36), SWLS ≤ 19 (38), GAD-7 ≥10 (31), PHQ-8 ≥5 (30), ESS >10 (32), and HIT-6 ≥50 (33)—based on previous reports (Table 1). Patients who scored at or above the threshold at admission for the specific measures were included in the analysis for that outcome measure (Figure 2). For the SWLS, scores within the range of 20–24 indicate general satisfaction; therefore, a cutoff of 19 or below was used to indicate significant dissatisfaction (38). The NSI does not have a composite threshold for symptomatic classification, and therefore an NSI change score was used.

### Data Analysis

Patient's response to treatment was defined as the difference between admission and discharge score for each assessment scale. The response was classified as clinically improved, improved, or did not improve. Clinically improved was defined as point changes between admission and discharge scores and based on available literature: NSI, ≥5-point change (34), PCL-M, ≥10-point change (37), SWLS, ≥5-point change (46), GAD-7, ≥5-point change (40), PHQ-8, ≥5-point change (42), ESS, ≥2-point change (44), and HIT-6, ≥8-point change (45) (Table 1). For the PCL-M, there is evidence that a five- to 10-point change represents a reliable change and a ≥10-point change represents a clinically significant change (37). The term improved relates to those showing a change in score for each scale that indicated recovery but did not reach a clinical threshold.

Assessment score normality was determined using the Shapiro–Wilks test and *Q*–*Q* plots. Nonparametric analysis was used to assess outcome differences at discharge and at 1, 3, and 6 months after discharge. Mean ranked differences on all measures were compared for score changes from admission to discharge and at 1, 3, and 6 months using a Wilcoxon signed-rank test. Bonferroni corrections were used to control for potential inflated family-wise error rates following multiple comparisons. The effect size of changes from admission was computed using the formula $r=Z/\sqrt{N}$ (47).

## Results

A total of 1,456 patients consented to the study (Figure 2). The study population was 98.4% male and 1.6% female, with a mean age of 38.3 years (SD = 7.1). The majority were members of the Navy (51.5%) and Army (30.6%) and had a mean service record of 17.3 years (SD = 7.0). The majority (90.0%) of SMs had a history of multiple TBIs (*M* = 7.0, SD = 8.3) (Table 2).

**Table 2:** Demographic data for the study population (*N* = 1,456).

**Variable**	**Value**
Age, years, *M* (SD)	38.3 (7.1)
Gender	
Male	98.4%
Female	1.6%
Ethnicity (*n* = 948)	
White	87.0%
Hispanic	4.2%
Black	4.0%
Asian or Pacific Islander	2.7%
American Indian or Alaskan	1.3%
Other	0.7%
Marital status	
Divorced	6.4%
Married	79.1%
Separated	4.0%
Single	10.4%
Widowed	0.1%
Years of service (*n* = 1,455), *M* (SD)	17.3 (7.0)
Number of deployments (*n* = 1,436)	
0	2.5%
1	6.6%
2 and 3	17.7%
≥4	73.2%
Branch of service	
Navy	51.5%
Army	30.6%
Marines	10.6%
Air force	7.2%
Coast guard	0.1%
Rank (*n* = 1,442)	
E-3, 4, 5, 6 E-7, 8, 9	79.6%
W-1, 2, 3, 4, 5 O-1, 2, 3, 4, 5, 6	20.3%
Number of TBIs (*n* = 1,373)	
*M* (SD)	7.0 (8.3)
Md	5.0
Quartile 1	≤3 (*n* = 453)
Quartile 2	4 and 5 (*n* = 314)
Quartile 3	6 and 7 (*n* = 209)
Quartile 4	≥8 (*n* = 397)
Mechanism of injury (*n* = 1,366)	
Blast	20.5%
Non-blast	14.8%
Mixed, blast, and non-blast	64.7%

*When information is unavailable for the entire population (N), a subset (n) is displayed*.

*y, year; M, mean; SD, standard deviation; Md, median; TBI, traumatic brain injury; E, enlisted; W, warrant officer; O, officer*.

**Figure 3:**
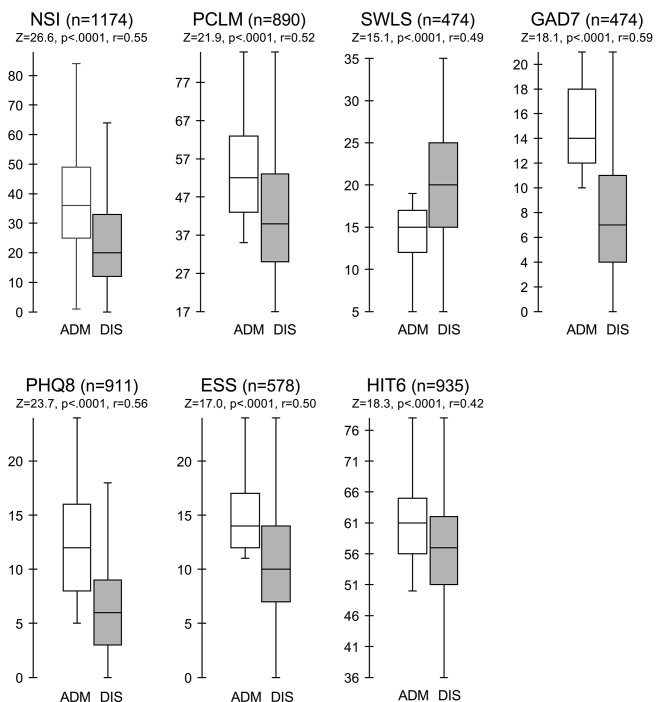
Comparison of assessment scores at admission and discharge for symptomatic patients. Box and whisker plots of assessment scores for symptomatic patients at admission (ADM, unfilled boxes) vs. discharge (DIS, shaded boxes). Boxes represent the interquartile (IQR) range with middle line indicating the median. Whiskers extend to 1.5 × IQR. Wilcoxon sign rank test comparing symptomatic patients at admission vs. discharge. *Significantly different from admission; Bonferroni correction *p* = 0.05.

Wilcoxon signed-rank tests revealed statistically significant improvements for symptomatic patients following treatment in the IOP for each of the seven assessments (Figure 3). The median score on the NSI decreased from admission (Md = 36) to discharge (Md = 20), *Z* = 26.6, *p* < 0.0001, with a large effect size (*r* = 0.55). The median score on the PCL-M decreased from admission (Md = 52) to discharge (Md = 40), *Z* = 21.9, *p* < 0.0001, with a large effect size (*r* = 0.52). The median score on the SWLS increased from admission (Md = 15) to discharge (Md = 20), *Z* = 15.1, *p* < 0.0001, with a large effect size (*r* = 0.49). The median score on the GAD-7 decreased from admission (Md = 14) to discharge (Md = 7), *Z* = 18.1, *p* < 0.0001, with a large effect size (*r* = 0.59). The median score on the PHQ-8 decreased from admission (Md = 12) to discharge (Md = 6), *Z* = 23.7, *p* < 0.0001, with a large effect size (*r* = 0.56). The median score on the ESS decreased from admission (Md = 14) to discharge (Md = 10), *Z* = 17.0, *p* < 0.0001, with a large effect size (*r* = 0.50). The median score on the HIT-6 decreased from admission (Md = 61) to discharge (Md = 57), *Z* = 18.3, *p* < 0.0001, with a large effect size (*r* = 0.42).

**Figure 4:**
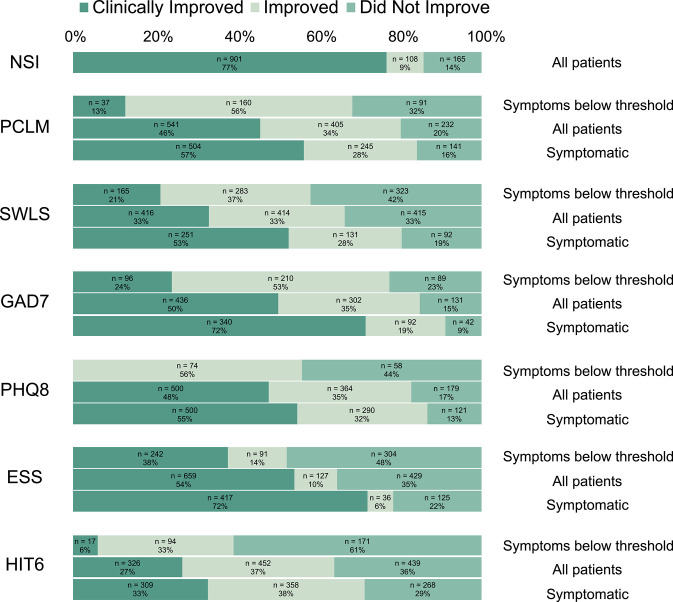
Clinical improvements following treatment in the NICoE 4-week intensive outpatient program. Clinical improvement was determined by the following point changes: NSI, ≥5 point: PCLM, ≥10 point: SWLS, ≥5 point: GAD7, ≥5 point: PHQ8, ≥5 point: ESS, ≥2 point: HIT6, ≥8 point. NSI does not have a composite threshold for clinically significant symptomatic classification. Symptomatic, Symptoms at or above threshold.

After treatment in the 4-week IOP, patients whose symptom severity was at or above threshold at admission showed clinical improvements at discharge in each of the seven assessments: NSI (77%), PCL-M (57%), SWLS (53%), GAD-7 (72%), PHQ-8 (55%), ESS (72%), and HIT-6 (33%) (Figure 4). In addition, an analysis of all patients, regardless of presenting symptom severity, showed improvements across each of the seven assessments (Figure 4; Supplementary Figure 1). For all assessments except HIT-6, clinical improvements were more likely to occur in patients with symptoms of the greatest severity.

Assessment scores from patients at 1, 3, and 6 months revealed the durability of outcomes (Table 3). The NSI median scores at admission were significantly higher (*p* < 0.0001) compared to patient-matched follow-up median scores at 1 month (Md = 22), 3 months (Md = 20), and 6 months (Md = 25), with large to medium effect sizes. The PCL-M median scores at admission were significantly higher (*p* < 0.0001) than the patient-matched median scores at 1 month (Md = 42), 3 months (Md = 42.5), and 6 months (Md = 46), with medium to small effect sizes. The SWLS median scores at admission were significantly lower (*p* = 0.0001) than the patient-matched median scores at 1 month (Md = 19), 3 months (Md = 20), and 6 months (*Md* = 19), with medium effect sizes. The GAD-7 median scores at admission were significantly higher (*p* < 0.0001) than the patient-matched median scores at 1 month (Md = 10.5), 3 months (Md = 9), and 6 months (Md = 11), with medium effect sizes. The PHQ-8 median scores at admission were significantly higher (*p* < 0.0001) than the patient-matched median scores at 1 month (Md = 7), 3 months (Md = 7), and 6 months (*Md* = 8), with medium effect sizes. The ESS median scores at admission were significantly higher (*p* < 0.0001) than the patient-matched median scores at 1 month (Md = 10), 3 months (Md = 11), and 6 months (Md = 12), with large to medium effect sizes. The HIT-6 median scores at admission were significantly higher (*p* = 0.0001) than the patient-matched median scores only at 1 month (Md = 58.5), with a small effect size.

**Table 3:** Wilcoxon signed-rank test of assessment scores at admission (ADM) vs. 1-, 3-, and 6-month time points for symptomatic patients.

**Measure**	***n***	**Time**	**Median**	***Z***	***P***	***r***
NSI	181	ADM	35	8.85	<0.0001^*^	0.47
		1 month	22			
	135	ADM	33	7.09	<0.0001^*^	0.43
		3 months	20			
	125	ADM	33	4.77	<0.0001^*^	0.30
		6 months	25			
PCL-M	133	ADM	50	6.93	<0.0001^*^	0.42
		1 month	42			
	82	ADM	47	3.12	0.0009^*^	0.24
		3 months	42.5			
	82	ADM	50	3.28	0.0005^*^	0.26
		6 months	46			
SWLS	71	ADM	14	5.64	<0.0001^*^	0.47
		1 month	19			
	46	ADM	15	3.8	0.0001^*^	0.40
		3 months	20			
	40	ADM	15	3.87	0.0001^*^	0.43
		6 months	19			
GAD-7	88	ADM	15	6.5	<0.0001^*^	0.49
		1 month	10.5			
	64	ADM	15	5.5	<0.0001^*^	0.49
		3 months	9			
	56	ADM	14.5	4.87	<0.0001^*^	0.46
		6 months	11			
PHQ-8	142	ADM	11	8.17	<0.0001^*^	0.48
		1 month	7			
	111	ADM	11	7.05	<0.0001^*^	0.47
		3 months	7			
	96	ADM	11	5.03	<0.0001^*^	0.36
		6 months	8			
ESS	76	ADM	14	6.21	<0.0001^*^	0.50
		1 month	10			
	67	ADM	14	5.67	<0.0001^*^	0.49
		3 months	11			
	64	ADM	14	4.51	<0.0001^*^	0.40
		6 months	12			
HIT-6	108	ADM	60	3.68	0.0001^*^	0.25
		1 month	58.5			
	95	ADM	59	2.63	0.0043	0.19
		3 months	58			
	89	ADM	61	1.5	0.0668	0.11
		6 months	61			

* *Significantly different from admission; Bonferroni correction p = 0.05. The median scores on ADM were calculated from only those who responded at each time point*.

The follow-up by electronic questionnaire was performed after discharge to obtain scores on the seven self-report scales (Supplementary Table 1). The average follow-up rate across all assessments was 15% at 1 month, 11% at 3 months, and 10% at 6 months. To address the concern that only those who had the best recovery would return the electronic follow-up self-report scales at 1, 3, and 6 months, the follow-up percent of patients who clinically improved during the 4-week IOP was compared to the follow-up percent of patients who did not have clinical improvement (Supplementary Table 1). No significant difference was found for any scale at any time point.

To test for potential selection bias in the longitudinal cross-section data, we compared the characteristics of those who responded to the follow-up self-report scales with those who did not respond at each of the three follow-up time points (Supplementary Table 2). Overall, the demographics of those who responded after discharge were comparable to the full study cohort at baseline. Notable exceptions are mean age, which was recorded at 40.5 (SD 6.4), 40.8 (SD 6.1), and 40.6 (Sd 5.7) at 1, 3 and 6 months, respectively, compared to 38.3 (SD 7.1) for all participants, mean years of service recorded at 19.5 years (SD 6.8), 20.1 years (SD 6.4), and 19.9 years (SD 6.0) at 1, 3, and 6 months, respectively, compared to 17.3 years (SD 7.0), and rank. As for rank, officers made up 20.3% of those completing the program and 25.6, 28.0, and 25.95% of those returning the longitudinal follow up surveys. at 1, 3, and 6 months, respectively. To determine if rank imparts a differential recovery pattern, analysis was performed to compare the longitudinal improvement between officers and enlisted personnel. The findings reveal that officers and enlisted personnel have similar recovery patterns of improvement in the NSI and PCL-M at 1, 3, and 6 months (Supplementary Table 3).

Additionally, the mean number of TBIs was significantly higher, mean of 8.6 (SD 11.1) and 8.9 (SD 12.8) at 1 and 6 months, respectively, compared to 7.0 (SD 8.3) TBI for all participants. Although modest in their difference, increased age, time in service, and number of TBIs would be anticipated to be associated with a less robust recovery. Thus, the current data support a positive impact of the IOP on symptom improvement durability even in a less favorable population. Furthermore, assessing recovery based on TBI exposure revealed that those SMs with a history of the greatest number of concussive events, quartile 4, had the greatest improvement in the NSI during the IOP (mean change of 15.46) compared to quartile 1 (mean change 12.47, *p* = 0.005). All other self-report indices had similar recovery patterns between the 1st and 4th quartiles (Supplementary Table 4).

## Discussion

In the military, exposure to blast and blunt force trauma and operational stressors from combat and mission training events related to the OEF/OIF conflicts resulted in an unprecedented number of military SMs presenting for treatment with these disorders. Despite conventional rehabilitation therapies, many SMs are deemed “unfixable” and forced to disengage from full active duty status or prematurely medically retire, thus reducing military readiness. To date, clinical practice guidelines have provided limited guidance for how to address these complex patients. This study demonstrates that an interdisciplinary IOP care model that combines conventional rehabilitation therapies with integrative medicine significantly improved the symptoms in patients with combat-related mTBI and comorbid PH conditions who were not responding to conventional therapies at the time of their referral to the NICoE. This study addressed three needed steps for advancing care in the TBI field: first, the applicability of delivering a complex treatment strategy over a sustained period in a large patient population; second, the need to address the simultaneous treatment of multiple domains; and third, the need to address the durability of recovery.

The program has cared for over 1,500 service members in all branches of the military, including both enlisted and officers, during the 7.5-year study period. Recovery was similar in all major groups of military service. The model of care significantly eliminates the fragmentation and reductionistic approach to medical care that challenges typical healthcare delivery in complex cases. The sustained care delivery and continuous benefits in each cohort demonstrate the feasibility as well as the efficacy of the model.

Findings from the study revealed a significant improvement across multiple clinical domains and importantly demonstrated durability. The Institute of Medicine (2014) recommends that research in the field of traumatic brain injury should emphasize on the clinical importance of using multiple validated rating scales to assess changes in co-morbid conditions (48). Our choice of measures assessed a broad range of symptoms across the psychological, physical, and cognitive domains. Future studies are needed to assess if there is a hierarchy of conditions that would dictate the optimal treatment sequences for a complex disease state. Furthermore, though a significant level of improvement across multiple domains was seen in most patients, some subjects failed to show an improvement in one or more of the scales (range from 9 to 19%). Additional work is needed to better understand differences in those that responded and those that did not.

The improvements noted in this study support that the care model results in sustained benefit. The 1-, 3-, and 6-month cross-sectional follow-up of patients after they returned to their duty station revealed a sustained improvement across most outcome measures, except headache at 3 and 6 months (Table 3). These findings support the durability of the benefits from this care model even after the SMs return to their prior work environment. Whereas, it may be speculated that a 4-week hiatus from the service members' work environment may have contributed to the initial recovery from admission to discharge, the brief departure alone is insufficient to account for the durability of the responses seen from discharge to 6 months. These changes are especially notable given the chronicity of the population. SMs referred to the NICoE reported months to years of persistent neurological and behavioral disturbances before attending the program.

In consideration of a potential selection bias in longitudinal assessment, the severity of symptoms was not different among the patients who responded and the entire cohort. Furthermore, our data support that those who were inclined to answer the follow-up questionnaires were older and had more TBIs. The SM with these characteristics would have been anticipated to have a less robust recovery response, and therefore the favorable outcomes in the follow-up data are likely to represent a meaningful recovery that can be extrapolated to the larger cohort.

The factors that contribute to the sustained improvement are postulated to be related to the foundational principles of the care model. Elimination of fragmented care through the interdisciplinary care plan and patient-centric team feedback that promotes rapid iterative treatment assessments could enhance the opportunity for multiple simultaneous domain improvements. The establishment of trust between the patient and care team and the improvement of sleep disturbances and pain as an initial program goal may provide the supportive non-judgmental environment and physiological restoration, respectively. This warrants further study. Although conventional psychotherapeutic treatments for PTSD were not used during the IOP, emphasis on integrative medicine techniques appeared to have a positive effect on BH conditions. Offerings such as art therapy, which was endorsed by SMs as extremely beneficial, are reported to leverage the externalization of the fragmented trauma narrative (25, 49), allowing the art therapist and other BH providers the ability to guide the patients' processing of the traumatic events, and will be the subject of future research. Programmatic emphasis on self-efficacy skills-based training and disease-specific educational module, also endorsed by SMs, may further contribute to longitudinal recovery strategies. Follow-up studies to identify which offerings were most helpful post-discharge are planned.

The data regarding the association of chronic effects of multiple TBI suggest that even SMs with a high number of exposures can experience improvement. Future study must include detailed trauma history and characterization to better understand the population risk and response to different treatment strategies.

Finally, in addition to the significant response to treatment, the program was extremely well-tolerated, with no patients leaving the 4-week program over the 7.5 years, a 100% completion rate. Only one of the 1,456 SMs withdrew his consent to be reached electronically after the program completed. This is in contrast to reported dropout rates of 36% or higher in conventional PTSD treatment (50). This intensive interdisciplinary integrative medicine paradigm is being adapted and implemented in 10 military treatment facility programs based on their individual patient's needs, staffing availabilities, and diagnostic capabilities. The program synchronization will provide a previously unavailable opportunity to continue to refine our understanding of mild TBI and comorbid PH conditions in active-duty SMs and assess the response to precision therapeutic strategies.

### Study Limitations and Way Forward

There are several limitations that provide guidance for future studies. This study uses a PBE analysis without a control group, owing to the real-world application of care, to not withhold the next level of treatment for SMs who were not on a trajectory of recovery despite conventional care. As a tertiary care facility program, the SMs were referred specifically due to the persistence or worsening of symptoms, and the study population had well-documented chronic neurological and psychological conditions prior to admission, arguing against spontaneous recovery. Future studies that leverage a wait list control group or use a variable model of treatment sequencing may provide greater confirmation of the program's efficacy and insight into the program's treatment strategies that produce the most benefit based on subpopulation presentation.

Another limitation is that the favorable response at follow-up could have been biased by the possibility that only those demonstrating a robust recovery would report their 1-, 3-, and 6-month scales. Based on their trajectory of recovery at discharge, we assessed the percent of patients responding at all follow-up time points for all self-report scales. The rate of returned SRSs was equivalent when comparing those who were clinically improved and those who were not at program discharge (Supplementary Table 1). Furthermore, though the study reveals a significant durability of outcomes up to 6 months, information regarding recovery at extended periods beyond that timeframe would be of significant utility. Also, since many of the skills-based techniques were endorsed by SMs during the IOP as beneficial, follow-up studies should also include information regarding patient endorsement of those treatments that help maintain a satisfactory recovery. Accessing patients by telephone follow-up, anticipated to obtain this type of information, was extremely limited due to the majority of SMs returning to high operational activity.

In addition, a more precise characterization of concussive event “dosing” would be helpful in identifying subpopulations for analysis of natural history and treatment response in the future. Number of events, blunt vs. blast vs. mixed concussions, timing of events, and physiological and emotional condition at the time of events can all play a role in brain injury severity. Reliable data collection strategies and models for analysis of these complex interaction remain a gap.

Given the benefits seen in the primary results of the study, future analyses to determine predictive factors from assessment modalities in the individual disciplines are being planned. Though advances in MR imaging are promising for identifying TBI changes, correlation with patterns definitive for chronic mTBI remains elusive. Future studies are planned for correlative assessment of MRI signal and specific injury pattern in this population.

Finally, though not specifically assessed in this study, cost modeling of an intensive interdisciplinary program is needed to assure the sustainability of this paradigm of care delivery. More recent constructs of an integrated practice unit have been explored as a model for disease-based care and warrant further exploration as a long-term strategy for programs that demonstrate significant clinical benefit (51).

## Conclusions

Our findings support that an interdisciplinary IOP that combines conventional rehabilitation therapies with integrative medicine techniques significantly improves a range of symptoms and holds promise for sustainable recovery among SMs suffering from co-morbid combat-related mTBI and PH conditions. These findings underscore the value of coordinated care delivery in complex brain injury and emphasize the importance of using symptom-specific outcome tools to assess efficacy in specific clinical subpopulations. Future studies should consider methodologies that lend themselves to identifying those active components of this rehabilitative care model that seem to account for the most variance in positive and sustainable treatment outcomes for service members with a history of mTBI and psychological health conditions.

## Data Availability Statement

The original contribution presented in the study are included in the article/Supplementary Material, further inquiries can be directed to the corresponding author.

## Ethics Statement

The studies involving human participants were reviewed and approved by Walter Reed National Military Medical Center Institutional Review Board (IRB). The patients/participants provided their written informed consent to participate in this study.

## Author Contributions

TJD, GN, and JB conceived and planned the study. TJD, RK, JLB, GN, and WP planned and carried out the treatment model. KW and TAD contributed to the analysis and designed the tables and figures. TJD, LF, TP, JK, GG, TAD, KW, JLB, WP, and JB contributed to the interpretation and writing of the manuscript. All authors contributed to the article and approved the submitted version.

## Conflict of Interest

TAD was employed by Booz Allen Hamilton. KW was employed by Credence Management Solutions. The remaining authors declare that the research was conducted in the absence of any commercial or financial relationships that could be construed as a potential conflict of interest.
