# Supplementary figures and images for: Efficacy of an Interdisciplinary Intensive Outpatient Program in Treating Combat-Related Traumatic Brain Injury and Psychological Health Conditions
Source: Front Neurol. 2021 Jan 18;11:580182. doi: 10.3389/fneur.2020.580182 (PMC7848806; doi:10.3389/fneur.2020.580182)

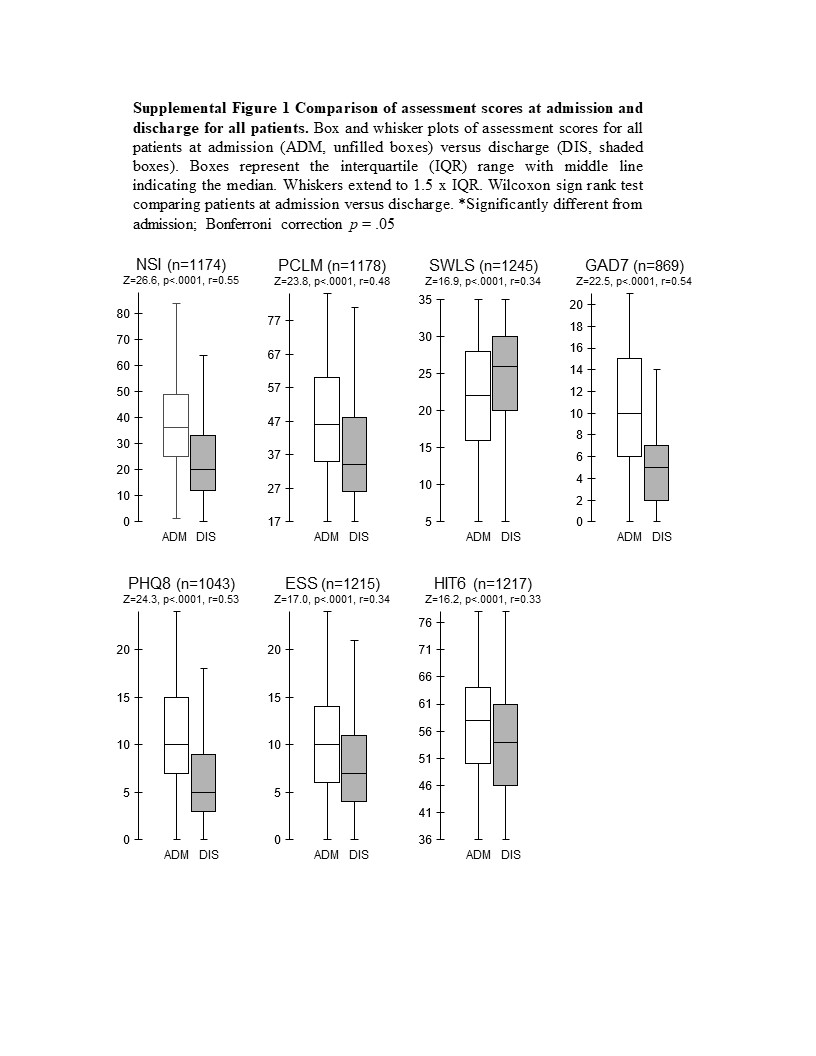

Supplement: Supplementary file 1 [file Image_1.JPEG]
