# Supplementary material for: Efficacy of an Interdisciplinary Intensive Outpatient Program in Treating Combat-Related Traumatic Brain Injury and Psychological Health Conditions
Source: Front Neurol. 2021 Jan 18;11:580182. doi: 10.3389/fneur.2020.580182 (PMC7848806; doi:10.3389/fneur.2020.580182)
Supplement: Supplementary file 2 [file Table_1.docx]

| **Supplemental Table 1.** To assess for the potential bias that only those with the best recovery replied to the electronic follow up self-report scales, the percentage of patients who clinically improved during the 4 week IOP that replied at 1,3 and 6 months was compared to those who did not clinically improve at discharge. No difference was seen in the follow-up rates of patients who clinically improved following treatment versus patients who did not experience a clinically significant improvement at discharge. P-values reflect chi-square tests. Bonferroni Correction *p* = .01 | | | | |
| --- | --- | --- | --- | --- |
| **Assessment** | **Discharge** | **1 Months** | **3 Months** | **6 Months** |
| **NSI** | Clinically improved  n=901 | 16%  n=143 | 12%  n=112 | 10%  n=93 |
|  | Did not clinically improve  n=273 | 14%  n=38 | 8%  n=23 | 12%  n=32 |
|  |  | *p=*.47 | *p=*.09 | *p=*.53 |
| **PCL-M** | Clinically improved  n=504 | 15%  n=75 | 8%  n=40 | 11%  n=54 |
|  | Did not clinically improve  n=386 | 15%  n=58 | 11%  n=42 | 7%  n=28 |
|  |  | *p=*.96 | *p=*.15 | *p=*.09 |
| **SWLS** | Clinically improved  n=251 | 15%  n=38 | 12%  n=29 | 8%  n=20 |
|  | Did not clinically improve  n=223 | 15%  n=33 | 8%  n=17 | 9%  n=20 |
|  |  | *p=*.92 | *p=*.17 | *p=*.71 |
| **GAD-7** | Clinically Improved  n=340 | 19%  n=64 | 14%  n=48 | 11%  n=39 |
|  | Did not clinically improve  n=134 | 18%  n=24 | 12%  n=16 | 13%  n=17 |
|  |  | *p=*.84 | *p=*.56 | *p=*.73 |
| **PHQ-8** | Clinically Improved  n=500 | 16%  n=80 | 12%  n=60 | 11%  n=56 |
|  | Did not clinically improve  n=411 | 15%  n=62 | 12%  n=51 | 9%  n=39 |
|  |  | *p=*.73 | *p=*.86 | *p=*.43 |
| **ESS** | Clinically Improved  n=417 | 13%  n=54 | 12%  n=50 | 11%  n=46 |
|  | Did not clinically improve  n=161 | 14%  n=22 | 11%  n=17 | 11%  n=18 |
|  |  | *p=*.83 | *p=*.65 | *p=*.96 |
| **HIT-6** | Clinically Improved  n=309 | 10%  n=30 | 9%  n=28 | 11%  n=34 |
|  | Did not clinically improve  n=626 | 12%  n=78 | 11%  n=67 | 9%  n=55 |
|  |  | *p=*.24 | *p=*.46 | *p=*.30 |
| **Average Follow-up Rate Per Time Point:** | | 15% | 11% | 10% |
