# Supplementary material for: Efficacy of an Interdisciplinary Intensive Outpatient Program in Treating Combat-Related Traumatic Brain Injury and Psychological Health Conditions
Source: Front Neurol. 2021 Jan 18;11:580182. doi: 10.3389/fneur.2020.580182 (PMC7848806; doi:10.3389/fneur.2020.580182)
Supplement: Supplementary file 3 [file Table_2.docx]

| **Supplemental Table 2. Comparison of study population demographics through follow up.** When information is unavailable for the entire population (*N*) a subset (*n*) is displayed. P-values reflect t-tests for continuous variables (Age, Years of Service, Number of TBIs) and chi-square tests for categorical variables (Gender, Ethnicity, Marital Status, Branch of Service, Rank, History of Multiple TBIs). For continuous variables, standard deviations are demarcated with parenthesis (SD). For categorical variables, the parentheses represent percentage (%). Abbreviations: y, year; *M*, mean; SD, standard deviation; Md, median; TBI, traumatic brain injury; E, enlisted; W, warrant officer; O, officer. Bonferroni Correction *p* = .001. | | | | | |
| --- | --- | --- | --- | --- | --- |
|  | **All patients**  **(baseline)** | **Symptomatic patients with ADM and DIS score** | **Symptomatic patients with ADM, DIS, and 1 MO score** | **Symptomatic patients with ADM, DIS, and 3 MO score** | **Symptomatic patients with ADM, DIS, and 6 MO score** |
|  | **N = 1,456** | **n = 1,271** | **n = 198** | **n = 146** | **n = 133** |
| **Variable** |  |  |  |  |  |
| Age, y, *M* (*SD*) | 38.3 (7.1) | 38.5 (7.0)  *p=*0.44 | 40.5 (6.4)  *p*<0.05 | 40.8 (6.1)  *p*<0.05 | 40.6 (5.7)  *p*<0.05 |
| Gender  Male  Female | 1,432 (98.4)  24 (1.6) | 1,251 (98.4)  20 (1.6)  *p*=0.83 | 195 (98.5)  3 (1.5)  *p*=0.88 | 144 (98.6)  2 (1.4)  *p=*0.79 | 130 (97.7)  3 (2.3)  *p=*0.58 |
| Ethnicity, n (%)  White  Hispanic  Black  Asian or Pacific Islander  American Indian or Alaskan  Other | n=948  825 (87.0)  40 (4.2)  38 (4.0)  26 (2.7)  12 (1.3)  7 (0.7) | n=815  711 (87.2)  37 (4.5)  30 (3.7)  22 (2.7)  10 (1.2)  5 (0.6)  *p=*0.99 | n=140  125 (89.3)  4 (2.9)  5 (3.6)  3 (2.1)  3 (2.1)  0 (0.0)  *p=*0.73 | n=93  85 (91.4)  1 (1.1)  3 (3.2)  1 (1.1)  3 (3.2)  0 (0.0)  *p=*0.22 | n=82  69 (84.1)  1 (1.2)  4 (4.9)  4 (4.9)  2 (2.4)  2 (2.4)  *p=*0.19 |
| Marital Status  Divorced  Married  Separated  Single  Widowed | 93 (6.4)  1,151 (79.1)  58 (4.0)  152 (10.4)  2 (0.1) | 83 (6.5)  1,001 (78.8)  55 (4.3)  131 (10.3)  1 (0.1)  *p=*0.94 | 9 (4.5)  167 (84.3)  12 (6.1)  10 (5.1)  0 (0.0)  *p*<0.05 | 8 (5.5)  116 (79.5)  8 (5.5)  14 (9.6)  0 (0.0)  *p=*0.86 | 5 (3.8)  118 (88.7)  4 (3.0)  6 (4.5)  0 (0.0)  *p=*0.09 |
| Years of service  *M* (SD) | n=1455  17.3 (7.0) | n=1270  17.6 (7.0)  *p=*0.19 | 19.5 (6.8)  *p*<0.05 | 20.1 (6.4)  *p*<0.05 | 19.9 (6.0)  *p*<0.05 |
| Branch of service  Navy  Army  Marines  Air Force  Coast Guard | 750 (51.5)  446 (30.6)  154 (10.6)  105 (7.2)  1 (0.1) | 671 (52.8)  379 (29.8)  125 (9.8)  95 (7.5)  1 (0.1)  *p=*0.83 | 109 (55.1)  59 (29.8)  12 (6.1)  18 (9.1)  0 (0.0)  *p=*0.24 | 93 (63.7)  36 (24.7)  7 (4.8)  10 (6.8)  0 (0.0)  *p*<0.05 | 80 (60.2)  35 (26.3)  4 (3.0)  14 (10.5)  0 (0.0)  *p*<0.05 |
| Rank  E-3, 4, 5, 6  E-7, 8, 9 | n=1442  1148 (79.6) | n=1257  1004 (79.9) | n=195  145 (74.4) | n=143  103 (72.0) | n=131  97 (74.0) |
| W-1, 2, 3, 4, 5  O-1, 2, 3, 4, 5, 6 | 294 (20.4) | 253 (20.1)  *p=*0.82 | 50 (25.6)  p=0.07 | 40 (28.0)  *p*<0.05 | 34 (26.0)  *p*=0.11 |
| Number of TBIs  *M,* (SD)  Md | 7.0 (8.3)  5.0 | 7.2 (8.6)  5.0  *p*=0.59 | 8.6 (11.1)  5.0  *p*<0.05 | 8.3 (11.0)  6.0  *p*=0.10 | 8.9 (12.8)  5.0  *p*<0.05 |
