# Supplementary material for: Efficacy of an Interdisciplinary Intensive Outpatient Program in Treating Combat-Related Traumatic Brain Injury and Psychological Health Conditions
Source: Front Neurol. 2021 Jan 18;11:580182. doi: 10.3389/fneur.2020.580182 (PMC7848806; doi:10.3389/fneur.2020.580182)
Supplement: Supplementary file 4 [file Table_3.docx]

| **Supplemental Table 3.** To assess potential differences in scores between enlisted and officer military ranks, change from Admission to 1, 3, and 6 months were compared. With the exception of PCL-M at 1 month, no significant differences in scores were seen between enlisted and officers for any of the longitudinal time points. P-values reflect Mann Whitney U tests. Bonferroni correction *p* = .01 | | | | | | | | |
| --- | --- | --- | --- | --- | --- | --- | --- | --- |
| **Assessment** | **Time Point** | **Rank** | **N** | **Median** | **Mean (SD)** | **U** | **Z** | ***p*** |
| **NSI** | 1 Month | Enlisted | 134 | 7.5 | 8.52 (11.23) | 2,400.5 | -1.85 | 0.065 |
|  |  | Officer | 44 | 12.0 | 12.84 (13.99) |  |  |  |
|  | 3 Month | Enlisted | 94 | 8.5 | 7.77 (11.72) | 1,398.0 | -1.95 | 0.051 |
|  |  | Officer | 38 | 11.5 | 12.76 (15.06) |  |  |  |
|  | 6 Month | Enlisted | 89 | 7.0 | 5.65 (13.56) | 1,482.0 | -0.175 | 0.861 |
|  |  | Officer | 34 | 6.5 | 6.71 (14.14) |  |  |  |
| **PCL-M** | 1 Month | Enlisted | 100 | 7.0 | 6.62 (11.96) | 974.5 | -2.905 | 0.004* |
|  |  | Officer | 30 | 15.0 | 14.87 (14.28) |  |  |  |
|  | 3 Month | Enlisted | 53 | 2.0 | 2.32 (13.05) | 508.5 | -1.884 | 0.060 |
|  |  | Officer | 26 | 9.5 | 9.12 (16.73) |  |  |  |
|  | 6 Month | Enlisted | 58 | 3.0 | 4.74 (14.15) | 573.0 | -0.701 | 0.483 |
|  |  | Officer | 22 | 5.0 | 5.77 (14.56) |  |  |  |
| Enlisted: E-3,4,5,6,7,8,9; Officer: W-1,2,3,4,5; O-1,2,3,4,5,6 | | | | | | | | |
