# Supplementary material for: Efficacy of an Interdisciplinary Intensive Outpatient Program in Treating Combat-Related Traumatic Brain Injury and Psychological Health Conditions
Source: Front Neurol. 2021 Jan 18;11:580182. doi: 10.3389/fneur.2020.580182 (PMC7848806; doi:10.3389/fneur.2020.580182)
Supplement: Supplementary file 5 [file Table_4.docx]

| **Supplemental Table 4.** To assess potential differences in self-report scale scores related to the number of TBIs a patient has sustained, the change from Admission to Discharge was compared for those in Quartile 1 vs Quartile 4 for each assessment. Apart from the NSI, no significant differences were seen in the amount a patient improved between Admission and Discharge for those who experienced the least and highest amount of TBIs in our sample population. P-values reflect Mann Whitney U tests. Bonferroni correction *p* = .01 | | | | | | | |
| --- | --- | --- | --- | --- | --- | --- | --- |
| **Assessment** | **Quartile** | **N** | **Median** | **Mean** | **U** | **Z** | ***p*** |
| **NSI** | Quartile 1 (≤ 3) | 335 | 11 | 12.47 | 40,672.0 | -0.301 | 0.005* |
|  | Quartile 4 (≥ 9) | 280 | 15 | 15.46 |  |  |  |
| **PCL-M** | Quartile 1 (≤ 3) | 292 | 11 | 11.49 | 33,924.5 | -0.793 | 0.428 |
|  | Quartile 4 (≥ 8) | 242 | 11 | 12.24 |  |  |  |
| **SWLS** | Quartile 1 (≤ 3) | 169 | 5 | 5.60 | 9,675.0 | -0.063 | 0.950 |
|  | Quartile 4 (≥ 8) | 115 | 5 | 5.46 |  |  |  |
| **GAD-7** | Quartile 1 (≤ 4) | 143 | 7 | 7.21 | 7,684.0 | -0.440 | 0.663 |
|  | Quartile 4 (≥ 10) | 111 | 7 | 7.04 |  |  |  |
| **PHQ-8** | Quartile 1 (≤ 3) | 229 | 5 | 5.07 | 25,247.5 | -0.530 | 0.596 |
|  | Quartile 4 (≥ 9) | 227 | 5 | 5.47 |  |  |  |
| **ESS** | Quartile 1 (≤ 3) | 175 | 4 | 3.26 | 10,379.0 | -2.43 | 0.015 |
|  | Quartile 4 (≥ 9) | 141 | 4 | 4.67 |  |  |  |
| **HIT-6** | Quartile 1 (≤ 3) | 291 | 4 | 4.73 | 36,406.0 | -0.301 | 0.764 |
|  | Quartile 4 (≥ 8) | 254 | 4 | 4.58 |  |  |  |
